# Beyond the patch: leveraging functional habitat delineation in fragmentation-biodiversity research

**DOI:** 10.1007/s10980-025-02290-y

**Published:** 2026-01-13

**Authors:** Matthew Dennis, Jonathan Huck, Claire Holt, Ewan McHenry, Erik Andersson, Sonali Sharma, Dagmar Haase

**Affiliations:** 1 CIRCLE, Department of Geography, University of Manchester, Manchester, UK; 2 Department of Science, Natural Resources & Outdoor Studies, University of Cumbria, Rydal Rd, Ambleside, UK; 3 Woodland Trust, Grantham, Lincolnshire UK; 4 Faculty of Biological and Environmental Sciences, Department of Environmental Sciences & Helsinki Institute of Sustainability Science, Helsinki, Finland; 5 Department of Geography, Lab for Urban Ecology, Humboldt University Berlin, Rudower Chaussee 16, 12489 Berlin, Germany; 6 Department of Computational Landscape Ecology, Helmholtz Centre for Environmental Research–UFZ, Leipzig, Germany; 7 Stockholm Resilience Centre, Stockholm University, Stockholm, Sweden

**Keywords:** Habitat delineation, Landscape models, Functional connectivity, Fragmentation, Biodiversity, Habitat amount

## Abstract

**Context:**

Theoretical and methodological developments in the field of fragmentation-biodiversity research continue to rely on the central concept of the habitat patch where patch size and number are considered particularly relevant to spatially structured ecological communities. However, although great interest has been shown in the effects of habitat fragmentation, appropriate methods for the spatial delineation of habitat have not received equal attention. In this paper, we argue that existing methods are not consistent with a functional definition of habitat as they fail to address key methodological challenges. These relate to the need to acknowledge a) the contribution of multiple resource types to habitat, b) the influence of neighbouring land cover types and c) the *continuity-contiguity problem* (the tendency of habitat to exhibit properties of gradation and aggregation).

**Objectives:**

In this second of two papers on this topic, we present an application of a new methodological framework outlined by Dennis et al. (this issue) that offers a route to a more functional definition and delineation of habitat through the use of spatial kernels and the generation of Type 1 and 2 fuzzy sets from landscape classification algorithms. We present a demonstration of the framework applied to a real-world landscape, in which we illustrate the impact of adopting alternative perspectives with respect to habitat delineation on the ecological process of habitat connectivity.

**Methods:**

We demonstrate the functional delineation of habitat for a focal generic woodland species (FGWS) in a real-world landscape classified through the application of a fuzzy Random Forest classifier. We employ nesting, foraging and dispersal parameters relevant to the FGWS to achieve a functional estimate of habitat. We test the influence of habitat fragmentation (number of patches controlling for total habitat amount) on potential functional connectivity for the FGWS based on contiguous (emphasising aggregation and homogeneity), continuous (emphasizing gradation) and functional (integrating multiple resource types and neighbourhood effects) habitat perspectives.

**Results:**

Our results indicate large discrepancies between the three perspectives on habitat delineation across key fragmentation-relevant metrics (total area, number of patches and potential functional connectivity). Importantly, a functional habitat perspective supports markedly different conclusions (compared to contiguous and continuous perspectives) with respect to the relationship between fragmentation (number of patches) and connectivity, and estimates of the contribution of individual habitat patches to landscape-scale connectivity.

**Conclusion:**

The functional habitat perspective, operationalized by harnessing uncertainty in landscape classification and employing spatial kernels to parameterise neighbourhood effects based on species-specific parameters, achieves a functional delineation of habitat. Our study suggests that such a view has major implications for our understanding of habitat fragmentation because it requires that the latter also be assigned a functional definition. The framework centred on functional habitat delineation is generalizable to a wide range of landscape contexts and advances current methods in spatial ecology. This opens up opportunities for inquiry and the development of new theoretical positions within the fragmentation-biodiversity debate.

**Supplementary Information:**

The online version contains supplementary material available at 10.1007/s10980-025-02290-y.

## Introduction

Current ideas in landscape ecology are heavily motivated by the need to address the major issues of habitat fragmentation and degradation and continue to be influenced by key theoretical concepts from island biogeography (MacArthur and Wilson 1967), the principles established by Forman (1992) and meta-population ecology (Hanski 1994). Theoretical and methodological developments in these areas rely on the central concept of the ‘habitat patch’ (Dennis et al. 2006), with the associated expectation that patch area is of particular importance for spatially structured ecological communities (Bosco et al. 2023). This assumption stems from some of the earliest ecological observations and related trends, principally the species-area relationship (Watson 1847). The species-area relationship was integrated into island biogeography theory as a supposed moderator of extinction and immigration rates thought to determine species turnover on islands (MacArthur and Wilson 1967). The resulting focus on patch size and isolation within an otherwise inhospitable matrix has prompted the adoption of spatial templates such as the patch-matrix-corridor model (Bogaert 2011) and related adages such as the “bigger, better more joined up” approach (Lawton 2010). Such models continue to carry weight in landscape management and decision making (Broome et al. 2025), asserting the centrality of the patch as the key unit of consideration in conservation and landscape planning.

However, the long-standing question around the influence of habitat formation, fragmentation and distribution on biodiversity outcomes (Valente et al. 2023) continues to divide opinion (Fletcher et al. 2018; Fahrig et al. 2019) and hypothesis testing within the field of fragmentation-biodiversity studies continues apace (Melo et al. 2017; Chase et al. 2020; Watling et al. 2020; Zhang et al. 2024; Watts and Hughes 2024; Riva et al. 2025). Despite the increasing attention paid to habitat fragmentation, the same momentum has not been reflected in the development of functional methods of habitat delineation, upon which our ability to measure fragmentation depends. In this paper we present methodological developments offering a route to a more functional definition (Dennis et al. 2014) and delineation (Dennis and Huck 2025; Dennis et al. this issue) of habitat that are highly relevant to fragmentation-biodiversity research.

This is the second of two papers addressing the inherent contradiction at the centre of habitat delineation which stems from the tendency of habitat resources to exhibit characteristics of both aggregation and gradation (the continuity-contiguity problem *sensu* Dennis and Huck (2025)). Dennis et al. (this issue) articulate the theoretical and practical challenges of working with the habitat patch concept and suggest a methodological framework that overcomes the continuity-contiguity problem and achieves a functional definition of habitat. In this paper we provide a demonstration of this framework applied to a case-study landscape and highlight the implications of adopting a functional approach to habitat delineation for fragmentation-biodiversity studies. We apply this method to the delineation of patches specifically within the context of fragmentation towards an understanding of how a functional view of fragmentation (based on our approach to patch delineation) can better inform research on the fragmentation-biodiversity relationship.

### The need for a functional delineation of habitat

Understanding causal factors in the distribution of species is of great relevance to the development of theory and practice in landscape ecology and related fields. Much recent research into this question has focused on the role of habitat fragmentation on biodiversity (Hanski 2015; Fletcher et al. 2018; Fahrig et al. 2019; Watts and Hughes 2024). With origins in species-area relationships, island biogeography and the SLOSS (single large or several small) literature, the debate as to whether fragmentation has an overall positive or negative influence on ecological communities has become known as the fragmentation-biodiversity debate (Valente et al. 2023). A key limitation obscuring hypothesis testing and theoretical development with respect to fragmentation effects stems from the tendency to base analysis on a raster representation of discrete land cover types, in which the landscape is a contiguous surface of square cells (pixels), each of which is classified as a single land cover type (Dennis and Huck 2025). To date, fragmentation-biodiversity studies have, to our knowledge, exclusively adopted a binary view of fragmentation, whereby each cell is considered as either habitat or not habitat (e.g. Evju and Sverdrup-Thygeson 2016; Lindgren and Cousins 2017; Melo et al. 2017; Chase et al. 2020; Watling et al. 2020; Zhang et al. 2024). This means that current theory on how fragmentation affects biodiversity at the landscape scale is driven by a structural (i.e. geometric) understanding of habitat and an area-based formulation (where the size of habitat polygons is assumed to be equal to resource availability), which may not reflect real-world processes.

These shortcomings are the result of conceptual and methodological challenges related to the patch concept, recently articulated as instances of the *continuity-contiguity problem* (Dennis and Huck 2025) which describes the intractability of accurately delineating patches (i.e. fragmentation) when habitat exhibits characteristics of both aggregation and gradation. This problem presents itself when either a binary representation of habitat (versus non-habitat with no intermediate classes) or a continuous representation of habitat (where different cover types intergrade and patterns of aggregation are ignored) is exclusively promoted. This over-arching problem perpetuates several methodological challenges related to the habitat patch concept: the gap-crossing problem (the confusion over whether to determine nearby habitat resources as a single or multiple patches); the need for multivariate habitat delineation (to reflect the use of multiple resources) and the problem of parameterising habitat as both a contiguous (spatially discrete) and continuous (intergrading) spatial entity. These problems obscure the relationship between fragmentation (i.e. number of patches), habitat heterogeneity and biodiversity. Key terms described above are explained further in Dennis et al. (this issue) and Dennis and Huck (2025) but summarized in Table 1.

**Table 1:** Definitions of habitat-related terms used in this study

Term	Definition
Contiguous habitat amount	The spatial extent of cells classified as habitat in a Boolean (binary) scheme where cells belong to one and only one class and the entire cell area is considered to be habitat
Continuous habitat amount	The sum of all cell values representing membership to the habitat class multiplied by the cell area
Multivariate habitat	Habitat delineation that involves summing cell membership-weighted habitat suitability values applied to cells of all cover types thought to represent habitat resources
Functional habitat amount	Multivariate habitat delineation that additionally accounts for (positive and negative) neighbourhood effects

We suggest that such challenges need not persist given recent developments in spatial-ecological methods of habitat delineation. In the first of this pair of papers, (Dennis et al. this issue) we present an approach to habitat definition-delineation that addresses the challenges related to the continuity-contiguity problem. This framework permits the navigation of theoretical and practical tensions implied by the tendency of habitat to exhibit properties of both aggregation and gradation and is compatible with landscape metrics that are widely-used in fragmentation-biodiversity analysis. This method draws on the integration of three key technical developments: 1. the leveraging of uncertainty in landscape classification algorithms, 2. the application of Monte Carlo resampling to achieve a broader appreciation of possible habitat composition and configuration outcomes for species of interest, and 3. the use of spatial kernels to operationalise positive and negative neighbourhood effects exerted by nearby cover types. This is an important step within fragmentation-biodiversity research. Though functional approaches to key landscape processes such as ecological connectivity have been promoted and developed before (Watts and Handley 2010; Dennis et al. 2014, Dennis et al. 2024a), a functional approach to habitat delineation remains absent from fragmentation studies. In this paper, we demonstrate how these problems can be addressed in the context of functional connectivity analysis through a real-world application of the method outlined in Dennis et al. (this issue).

## Methods: a simple, real-world case study

**Fig. 1:**
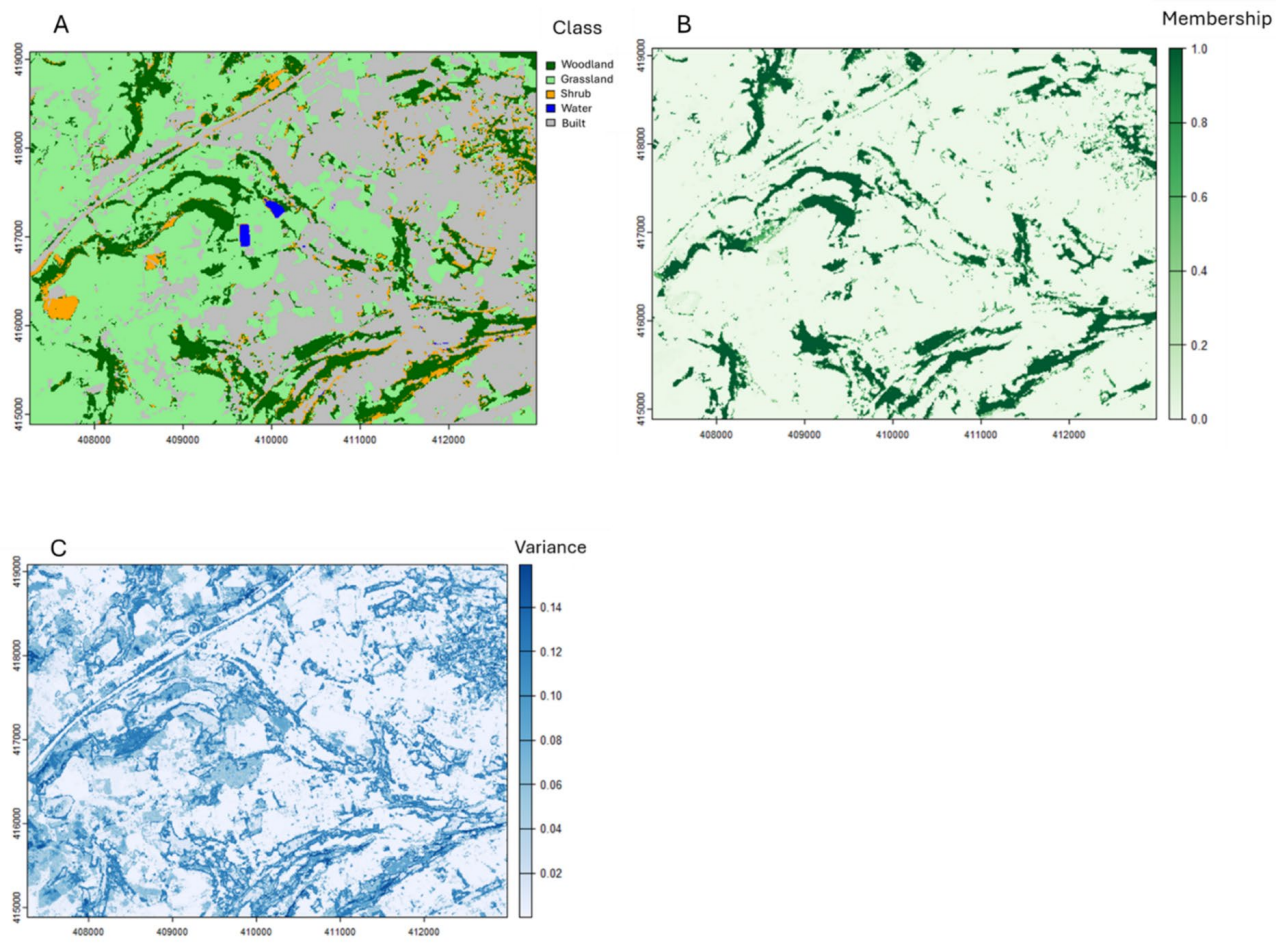
Landscape produced with a Random Forest classifier depicting **A** Boolean membership, **B** *Type-1* fuzzy membership, and **C** Uncertainty (model variance)

We demonstrate our approach on the landscape depicted in Fig. 1 (Location: Colne Valley, West Yorkshire, UK (latitude = 53.620, longitude =  − 1.882). The representation in Fig. 1A shows a Boolean land cover scheme resulting from a Random Forest classifier applied to a 9 m resolution colour infra-red image (Planet Labs PBC 2024). The model was trained on ~ 4500 sampling points and exhibits an overall accuracy of 95%. The study area comprises ~ 15% woodland cover that is primarily associated with the riparian and grassland areas to the West and increasingly with urban land-use moving Eastward. We focus on land cover here for two reasons. Firstly, because the current biodiversity crisis is inextricably associated with the loss and fragmentation of biodiversity-supporting vegetation complexes (Evju and Sverdrup-Thygeson 2016; Lindgrem and Cousins 2017; Melo et al. 2017; Watling et al. 2020), we focus on land cover types representing vegetation. Secondly, although abiotic conditions may also contribute to habitat resource distributions, such contributions can be delineated by adopting an appropriate classification scheme (for example using training data for wet woodland in addition to wetland and woodland) or delineated in a continuous way when membership to multiple classes (e.g. water, woodland) is permitted through multivariate habitat delineation (Sect. “Habitat delineation approach” and see also Dennis et al., this issue).

The study area is typical of a modern fragmented agricultural landscape with a mix of non-habitat land-uses and has been used elsewhere to effectively study the effects of fragmentation on biodiversity outcomes (Dennis et al. 2024b). Figure 1B shows the membership of each cell to the woodland class based on the mean prediction for this class from the Random Forest classifier and Fig. 1C shows the variance around these values.

### Habitat delineation approach

From the landscape information given in Fig. 1, we delineated habitat and subsequently computed potential functional connectivity for a focal generic woodland species (FGWS). The FGWS was parameterized according to nesting and foraging values associated with different land covers taken from Gardner et al. (2024, Supplementary Materials Table S1) for specialist woodland birds. For estimates of effective distance and matrix impacts, values relating to movement cost and edge effects for the same landscape (from a study by Eycott et al. (2011), Supplementary Materials Table S2) were used. Note that, whilst we use parameters from other studies here, these values can equally be determined empirically. For example, a species distribution modelling approach could be employed to understand species-environment associations. We suggest, however, that associations should be based on *type-1* membership to each cover type and modelled through *type-2* fuzzy classification (Sects. Landscape simulation approach; see also Dennis et al. this issue), thus avoiding the continuity-contiguity problem. In both cases, uncertainty associated with such values, if known, can also be incorporated into our approach by representing them as random variables in the Monte Carlo Analysis.

Habitat delineation was carried out following three steps. First, values for nesting suitability for the FGWS were used to assign weights to land covers in the study area then, for each location (raster cell), habitat suitability was calculated as the weighted sum of all land covers making a potential contribution to nesting habitat. Each cell’s contribution to habitat ($$habitat_{c} )$$ is then given as:

1 $$habitat_{c} = \mathop \sum \limits_{{\text{i = 1}}}^{{\mathrm{|M|}}} M_{ci} H_{i}$$

Where $M$ is the set of membership values to each land cover class $i$ for the current cell, ${\mathrm{|M|}}$ is the length of that set, $H$ is the set of the corresponding habitat suitability values for each land cover class, and $M_{ci}$ and $H_{i}$ are the membership value of cell *c* to land cover class *i*, and the habitat suitability value for land cover class *i*, respectively. Once habitat value (range: 0–1) is computed for each cell, an $\alpha$-cut (alpha cut) of 0.5 is used to determine habitat from non-habitat cells and individual patches delineated as groups of adjacent cells using a queen neighbourhood rule (i.e. eight nearest neighbours).

In the third step, we apply the foraging scores from Gardner et al. (2024) to each land cover class to assess the contribution of nearby resources to the delimited habitat cells. The contribution of each cell to habitat as a function of foraging suitability ($forage_{c}$) was computed as:

2 $$forage_{c} = \mathop \sum \limits_{{\text{i = 1}}}^{{\mathrm{|M|}}} M_{ci} F_{i}$$

Where $M$ is the set of cell membership values to each land cover class *i* for the current cell $c$, ${\mathrm{|M|}}$ is the length of that set, $F$ is the set of the corresponding foraging suitability values, and $M_{ci}$ and $F_{i}$ are the membership values of cell *c* to land cover class *i*, and the foraging suitability value for land cover class $i$, respectively. The likelihood of secondary resources being accessible from habitat cells was computed as a function of their distance from the nearest habitat cell and the maximum foraging distance of the FGWS in Gardner et al. (2024) by applying a negative exponential kernel to each cell’s distance from the nearest habitat cell. We refer to the likelihood of any cell as having an effect on another cell within its neighbourhood as $P_{ck}$ which is calculated as:

3 $$P_{ck} = (e^{{ - \alpha D_{ck} }} )$$

Where *e* is the natural exponent, $D_{ck}$ is the distance between cell *c* and neighbouring cell *k* and $\alpha$ is a constant that determines the probability of effect (in this case, the likelihood of foraging resources being accessible) at distance $c$. Here we set alpha to reflect the probability of movement to a cell at the maximum foraging distance of the FGWS being 0.05. This is determined using the following equation:

4 $$a = \frac{{ - {\mathrm{log}}\left( d \right)}}{maxD}$$

Where $maxD$ is parameterized according to the maximum foraging distance of the FGWS and $d$ is a distance decay parameter set to 0.05 reflecting the probability of movement to a cell at $maxD$.

In the case of positive neighbourhood effects, we computed $D_{ck}$ based on the functional cost of movement between cells. Functional cost is derived for each cell as the sum of membership-weighted cost values for all land-cover classes, plus an additional cost incurred as a function of negative neighbourhood effects from nearby land covers. This reflects the mechanistic understanding that movement paths within suitable habitat but in close proximity to land covers of high cost (for example, built infrastructure) may be less attractive than paths through less suitable habitat but which occur within a relatively less hostile background matrix. To compute functional cost, we assigned cost values of 1, 10, 2, 10 and 5 to woodland, grassland, shrub, water and urban land-cover respectively according to values used in a similar context by Eycott et al. (2011). Additional cost for each cell was then estimated as a function of negative neighbourhood effects whereby cells are assigned greater cost as a function of their proximity to deleterious land cover. In this case we consider grassland and urban classes to exert a negative neighbourhood effect (Eycott et al. 2011) and compute $P_{ck}$, setting component $a$ (as per Eq. 4) such that $maxD$ reflects the edge effect distance for each land cover (see also Eq. 7/Sect. “Parameterization of negative neighbourhood effects on habitat”) and the distance decay parameter $d$ is set according to the cell size (Dennis et al. 2024a). The functional cost of cell $c$ is then given as:

5 $$functionalCost_{c} = \mathop \sum \limits_{{\text{i = 1}}}^{{\mathrm{|M|}}} M_{ci} R_{i} + \mathop \sum \limits_{{\text{k = 1}}}^{{{|K|}}}\mathop \sum \limits_{{\text{i = 1}}}^{{{|M_k|}}} P_{ck} M_{ki} R_{i}$$

Where $M$ is a set of the cell membership values to each land cover class for the current cell *c*, $R$ is the set of the corresponding resistance values, $M_{ci}$ is the membership to class $i$ for the current cell $c$, $R_{i}$ is the resistance value for class $i$, *K* is the set of cells in the neighbourhood of cell *c* assumed to exert a neighbourhood effect, *M*_*k*_ is the set of land cover class membership values for neighbouring cell *k*, *P*_*ck*_ is the likelihood of cell *k* having a neighbourhood effect on cell *c* (as per Eq. 3 and 4), and *M*_*ki*_ is the membership value of neighbouring cell *k* to land cover class *i.* The final positive neighbourhood effect exerted by neighbouring  cells on habitat cell $c$ is then:

6 $$nPos_{c} = \frac{{\mathop \sum \limits_{{\text{k = 1}}}^{{{|K|}}}\mathop \sum \limits_{{\text{i = 1}}}^{{{|M_k|}}} P_{ck}M_{ki} F_{i} }}{\left| C \right|}$$

Where *C* is the total number of cells within the foraging distance of habitat cell $c$, *K* is the set of cells in the neighbourhood of cell *c* assumed to exert a neighbourhood effect, *M*_*k*_ is the set of land cover class membership values for neighbouring cell *k*, $P_{ck}$ is the likelihood of neighbouring cell *k* exerting a neighbourhood effect on cell $c$,  parameterized by measuring $D_{ck}$ as functional cost and setting $maxD$ as the foraging range of the FGWS, *M*_*ki*_ is the membership of neighbouring cell *k* to land cover class *i*, and *F*_*i*_ is the foraging suitability value for land cover class *i*. 

### Parameterization of negative neighbourhood effects on habitat

Negative neighbourhood effects on habitat incurred by the surrounding matrix were estimated via a negative exponential kernel as per Eq. 3 and 4 and the calculation of functional cost but where habitat is removed from habitat cell $c$ as a function of its distance from land covers exerting a negative neighbourhood effect. This negative neighbourhood effect $nNeg_{c}$ exerted on each habitat cell $c$ is then:

7 $$nNeg_{c} = \mathop \sum \limits_{{\text{k = 1}}}^{{{|K|}}}\mathop \sum \limits_{{\text{i = 1}}}^{{{|M_k|}}} P_{ck} M_{ki}$$

Where $K$ is the set of the cells in the neighbourhood of cell *c* assumed to exert a neighbourhood effect, *M*_*k*_ is the set of land-cover class membership values  for neighbouring cell *k*, *P*_*ck*_ is the likelihood of neighbouring cell *k* exerting a neighbourhood effect on cell *c*, and $M_{ki}$ is the membership of neighbouring cell *k* to land cover class $i$. The final estimate of functional habitat $fHab_{c}$ is then obtained as:

8 $$fHab_{c} = habitat_{c} + nPos_{c} - nNeg_{c}$$

For the purposes of using $fHab_{c}$ (or $habitat_{c}$) in subsequent patch-based analyses, the total habitat amount within a delimited patch is then the sum of all $fHab_{c}$ values for cells within a patch multiplied by the cell size. We refer to the patch-level habitat value (for use in the patch-based connectivity assessment: Eq. 9) as $pHab$. 

**Fig. 2:**
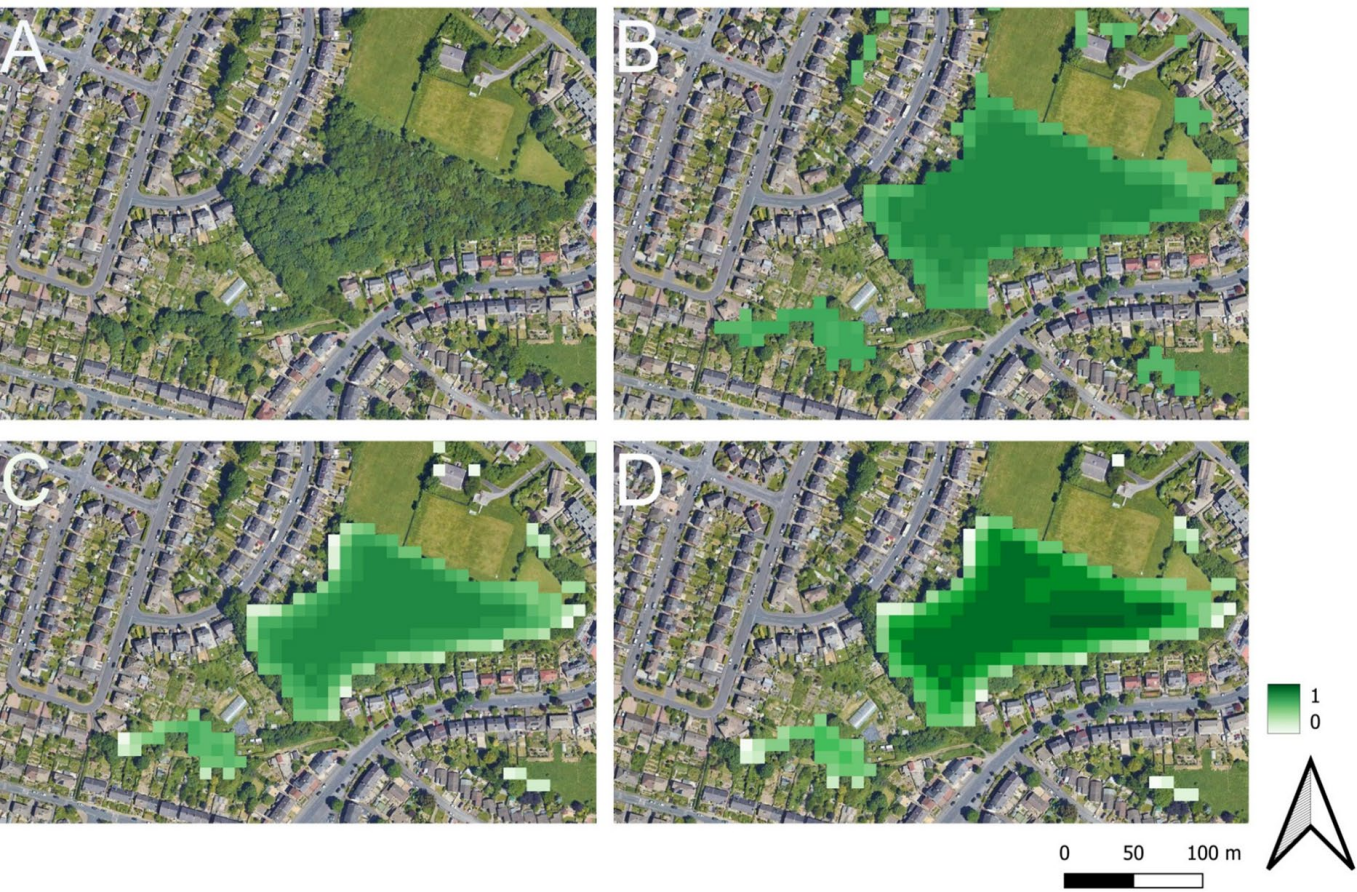
Habitat estimates for **A** a habitat patch in the study area landscape, **B** multivariate patch delineation with an $\alpha$ cut of 0.5 applied (no neighbourhood effects), **C** multivariate patch delineation with an $\alpha$ cut of 0.5 after negative neighbourhood effects considered, and **D** functional habitat: multivariate patch delineation with an $\alpha$ cut of 0.5 applied after considering positive and negative neighbourhood effects Maps data: Google © 2025

Figure 2 gives an example woodland patch in the study area landscape (Fig. 2A) and its determination as multivariate continuous habitat (i.e. $habitat_{c}$; Fig. 2B), continuous habitat considering only negative neighbourhood effects (Fig. 2C) and functional habitat ($fHab_{c}$; Fig. 2D). Note that the functional approach (Fig. 2D), which includes negative and positive neighbourhood effects, exhibits the broadest range of habitat values. This reflects the fact that urban land cover is deemed to exert an edge effect whilst also containing foraging resources (e.g., provided by the residential gardens visible in Fig. 2) in the parameters taken from Gardner et al. (2024) and Eycott et al. (2011). If a species’ foraging range is sufficiently large, then the ability to access nearby resources may therefore offset negative neighbourhood effects, even from the same land cover type. If the foraging range is greater than the maximum negative neighbourhood effect, this can logically result in much lower functional habitat values at the edge of patches relative to the core. This is because habitat cells towards the centre of the patch may escape large negative neighbourhood effects whilst still having their value uplifted as a function of foraging potential. This effect can be seen in Fig. 2D, where values towards the patch edge are lower but values towards the patch interior are higher than the corresponding cells in the continuous habitat scenario in 2B. This demonstrates how operationalising a functional perspective can offer a more refined estimate of habitat availability than a purely contiguous or continuous view.

### Calculating potential functional connectivity

We calculated the potential functional connectivity of the study area landscape based on the edge-weighted habitat index approach (EHI, Dennis et al. 2024a). The EHI is a graph-theoretic connectivity metric that returns the amount of connected habitat (as a percentage of the total landscape area) after accounting for landscape permeability and edge effects. Previously, the EHI has only been used to integrate negative edge effects into connectivity assessments. Hence, here we update the EHI approach by considering both positive and negative influences of nearby land-cover as an overall neighbourhood effect. The use of functional cost represents another update to the EHI metric. We refer to the metric returned by this updated approach incorporating neighbourhood effects and functional cost as the reachable functional habitat (RFH) and is computed as:

9 $$RFH = { }\frac{{\sqrt {\sum_{{p = 1{ }}}^{n} \sum_{{q = 1{ }}}^{n}  pHab_{p} A pHab_{q}A{ }P_{pq}^{*} } }}{{A_{L} }} \times 100$$

Where: $pHab_{p}$ and $pHab_{q}$ are the sum of habitat values within patches $p$ and $q$, $A$ is the cell area, $A_{L}$ is the total landscape area (i.e. study extent), and dispersal probability between patches $p$ and $q$ is defined as the maximum probability of movement (where $P_{pq}^{*}$ is the maximum product probability of all the possible paths between patches $p$ and $q$) based on shortest paths in a probabilistic patch-based graph. The result is multiplied by 100 to render the metric as a percentage.

**Fig. 3:**
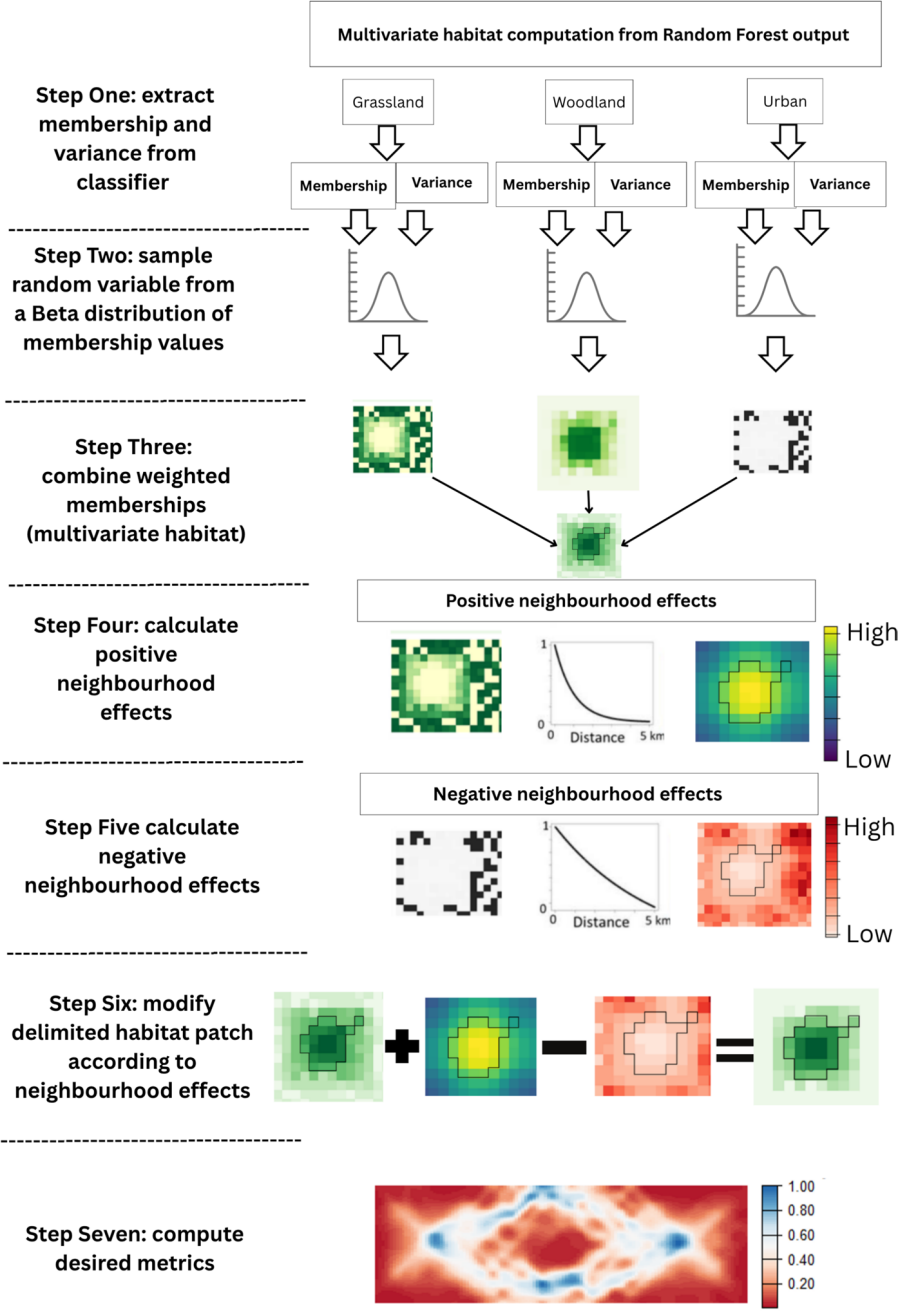
Overview of steps describing the workflow used in this study: Step 1. Extract membership and variance from classifier; Step 2 sample random value from a Beta distribution of possible membership values; Step 3. Calculate multivariate habitat from habitat suitability-weighted membership for each class; Step 4. Calculate positive neighbourhood effects; Step 5. Calculate negative neighbourhood effects; Step 6 Combine multivariate habitat with neighbourhood effects; Step 7. Computation of fragmentation-sensitive patch metrics

Figure 3 gives an overview of the entire work flow in this study split into seven steps: 1. Extracting membership and variance from the classifier; 2. Sampling membership to all classes for each cell from a Beta distribution of possible values based on the Random Forest output; 3. Calculating multivariate habitat from habitat suitability-weighted membership to each class; 4. Calculation of positive neighbourhood effects; 5. Calculation of negative neighbourhood effects; 6. Combining multivariate habitat with neighbourhood effects; 7. Computation of fragmentation-sensitive patch metrics (connectivity in this study).

### Landscape simulation approach

Dennis et al. (this issue) describe the use of Monte Carlo simulation based on a *type-2* fuzzy surface of land cover membership derived from the uncertainty inherent in the underlying classification model (Random Forest, in this case). This provides a means to parameterise habitat as both a discrete and continuous spatial entity in fragmentation-biodiversity studies. Dennis et al. (this issue) describe and illustrate the method using a small set of simulated patches. Here we demonstrate how the approach can be applied at the landscape scale. When calculating random landscape scenarios from a *type-2* fuzzy surface (i.e., a surface of possibility distributions) at the landscape scale, the Common Random Numbers (CRN) technique (Schruben 1978) should be used to avoid underrepresenting membership uncertainty. In its simplest form, CRN simply requires that multiple systems are simulated using the same set of random numbers to permit comparison between simulation-based models (Glasserman and Yao 1992). For example, CRN was used across multiple variables by Cyr et al. (2017) in their assessment of the effect of harvesting activities on caribou habitat to ensure that random variables relating to wildfires (i.e., date, location, size) were shared between different simulation scenarios. In the case of a *type-2* fuzzy landscape, each cell comprises a Beta distribution of values from which a random variable is drawn to create a given landscape scenario (i.e., the landscape model is made up of a surface of random variables). The goal of CRN in this context is therefore to ensure consistency between all of the random variables in a given landscape, as opposed to drawing a new random value separately for each individual cell, which would reduce the variability between scenarios and so underrepresent the uncertainty in the analysis. For each iteration of the Monte Carlo simulation, the landscape scenario is determined using a single random value $x$ drawn from a standard uniform distribution. $x$ is then used to determine the membership value of each individual cell using the percent-point function $ppf\left( x \right)$ of its Beta distribution, such that all membership values in a given landscape scenario represent the membership value for that cell with possibility $P\left( x \right)$.

For potential functional connectivity, we then computed the RFH metric (Eq. 9) according to three different perspectives with respect to habitat delineation: **1** a multivariate contiguous approach (*sensu* Dennis and Huck 2025) to the delineation and quantification of habitat (where cells with $habitat_{c}$ ≥ 0.5 contribute their entire area to habitat), **2** a multivariate continuous approach to habitat (where cells with $habitat_{c}$ ≥ 0.5 contribute their cell area multiplied by their $habitat_{c}$ values) and **3** the functional habitat approach. The single value obtainable from a Boolean perspective was also computed for comparison. Calculating connectivity from a multivariate contiguous perspective does not consider the role of habitat-matrix transitions nor neighbourhood effects. It is equivalent to computing the Probability of Connectivity (PC) metric (Saura and Pascual-Hortal 2007) on patches resulting from step three in Fig. 3 where the geometric area of patches formed of adjacent cells with $habitat_{c}$ ≥ 0.5 contributes to habitat amount. Connectivity based on a multivariate continuous perspective (acknowledging habitat-matrix transitions) is equivalent to computing PC where habitat amount in each patch is the sum of $habitat_{c}$ values ≥0.5 multiplied by the cell area but without parameterizing neighbourhood effects (also equivalent to step three in Fig. 3). Finally, the functional habitat approach integrates both habitat-matrix transitions and neighbourhood effects into the RFH metric. The key difference between these three approaches stems from the quantification of habitat amount and the emphasis placed on neighbourhood effects. In all three scenarios, a new landscape is generated at each iteration, and habitat patches are then delineated via an alpha cut of 0.5, resulting in a different habitat amount that is entered into the functional connectivity metric. In the case of the multivariate contiguous approach, the area of the entire delimited patch is used as an estimate of habitat amount. In the multivariate continuous approach, the sum of the $habitat_{c}$ values of cells within the patch ($pHab$) are used multiplied by the cell area. The same approach is used in the case of functional habitat amount but where $pHab$ is the sum of all $fHab_{c}$ values within the patch. For clarity we refer to the functional connectivity measure in scenarios 1 and 2 as RH (reachable habitat) and in scenario 3 as RFH (reachable functional habitat). At each iteration, we also computed the relative influence of each patch arising in the simulation on potential functional connectivity. We achieved this by computing $dRH$ (difference in reachable habitat) for each patch. $dRH$ is the change in the connectivity metric caused by that patch being removed from the analysis.

Each of the three perspectives was explored by the computation of the respective metric for 1000 values of $x$. We explored the influence of each perspective on potential functional connectivity estimates by comparing the distribution of values produced and formally tested the relationship between fragmentation *per se* and functional connectivity through regression analysis. Here, using the simulation results for each scenario ($n$ = 1000), we entered the number of delimited patches in the landscape and the total habitat amount as predictor variables and the respective functional connectivity metric as the response variable into a generalised linear model (GLM) with a Gaussian error distribution. In the multivariate contiguous approach, total habitat amount is taken to be the sum of the area of all cells with $habitat_{c}$ values above the $\alpha$ cut (i.e. the entire cell area contributes to habitat amount) and, in the continuous approach, habitat amount is the sum of all $habitat_{c}$ values above the $\alpha$ cut multiplied by the cell area (i.e. cell area is weighted by $habitat_{c}$). The same logic is used to compute functional habitat amount but where $fHab_{c}$ replaces $habitat_{c}$.

## Results

**Fig. 4:**
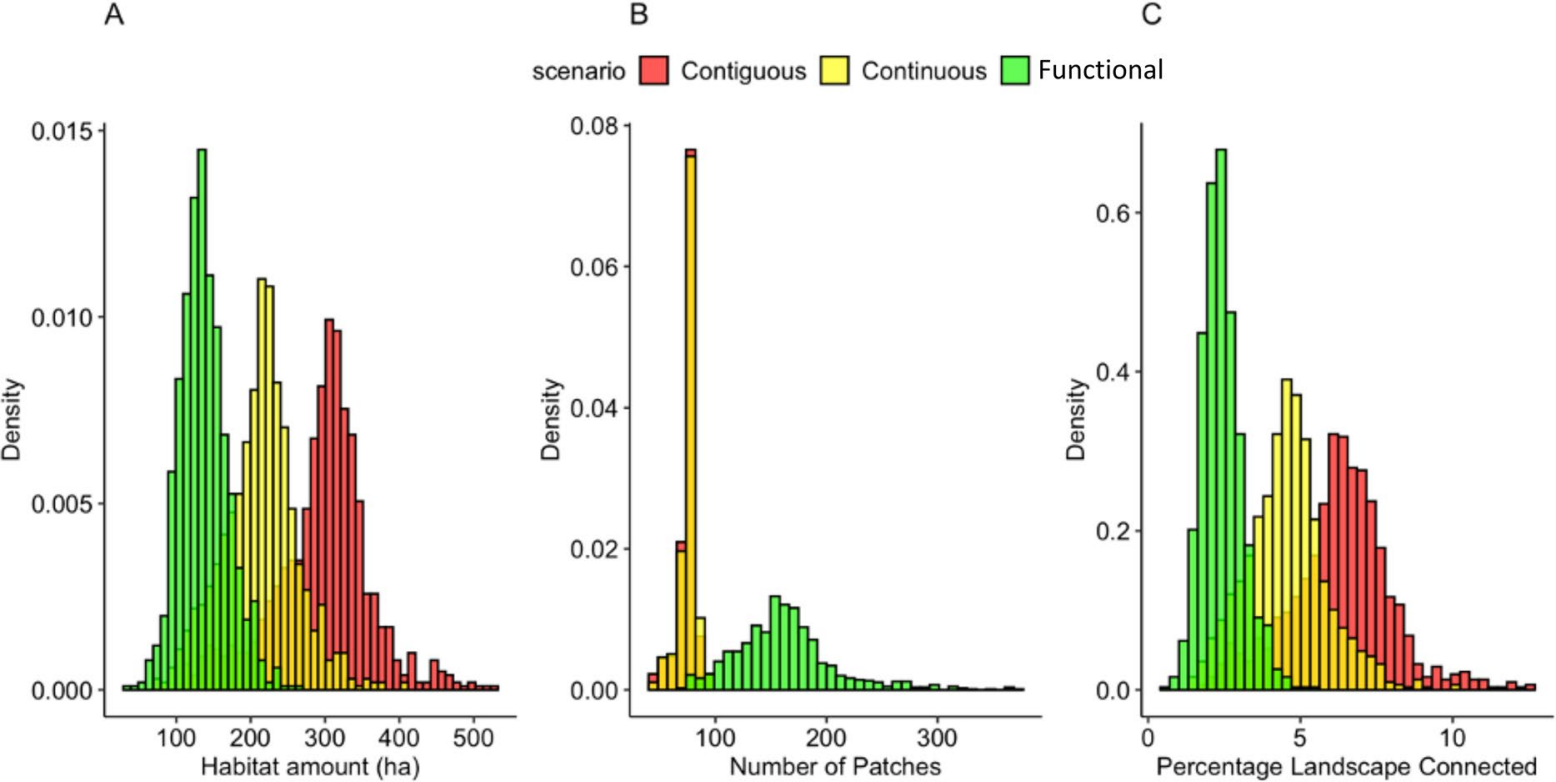
Distribution of **A** habitat amount, **B** number of patches, and **C** functional connectivity estimates for the study area landscape; according to the three habitat delineation scenarios: multivariate contiguous, multivariate continuous and functional

The distributions related to habitat amount, number of patches and potential functional connectivity varied significantly between the different perspectives towards habitat delineation (Fig. 4). In all cases, the mean connectivity value was considerably lower than that calculated for the Boolean landscape. Table 2 gives the minimum, mean and maximum estimates of functional connectivity based on the multivariate contiguous, multivariate continuous and functional perspectives and the single estimate obtained for the Boolean landscape shown in Fig. 1A.

**Table 2:** Summary of connectivity estimates for multivariate contiguous, multivariate continuous, functional, and Boolean approaches to habitat delineation for the study area landscape

	Min	Mean	Max	Variance
Boolean	–	9.08	–	–
Multivariate contiguous	1.83	6.32	12.55	2.82
Multivariate continuous	1.36	4.56	10.13	1.70
Functional	0.55	2.42	5.42	0.43

Boolean refers to a single assessment of the connectivity based on the classification scheme in Fig. 1A applying the probability of connectivity metric (Saura, S. and Pascual-Hortal 2007)

The highest prediction for potential functional connectivity occurs in the multivariate contiguous delineation of habitat. By contrast, when neighbourhood effects are considered (functional habitat), this maximum is reduced by almost half (Fig. 4C). This demonstrates how a Boolean view of habitat patches (and, more precisely, habitat cells contributing to patches), ignores a potentially large amount of variation at the landscape-scale. It also suggests that opting to ignore the influence of neighbourhood effects may significantly over-estimate the proportion of the landscape that is effective (functional) habitat, particularly in landscapes with high contrast transitions between areas of habitat and neighbouring deleterious land-uses. The associations between fragmentation *per se* (number of patches controlling for habitat amount) and connectivity in the GLM analysis are given in Table 3, indicating that the strength of this relationship weakens with increasing emphasis placed on a functional view of habitat.

**Table 3:** Beta coefficient estimates from the GLM analysis showing the strength of association between number of patches in the landscape and potential functional connectivity (controlling for habitat amount)

Model	Beta	*P* Value
Multivariate contiguous	−0.04357	< 0.001
Multivariate continuous	−0.03112	< 0.001
Functional	0.005486	0.173

**Fig. 5:**
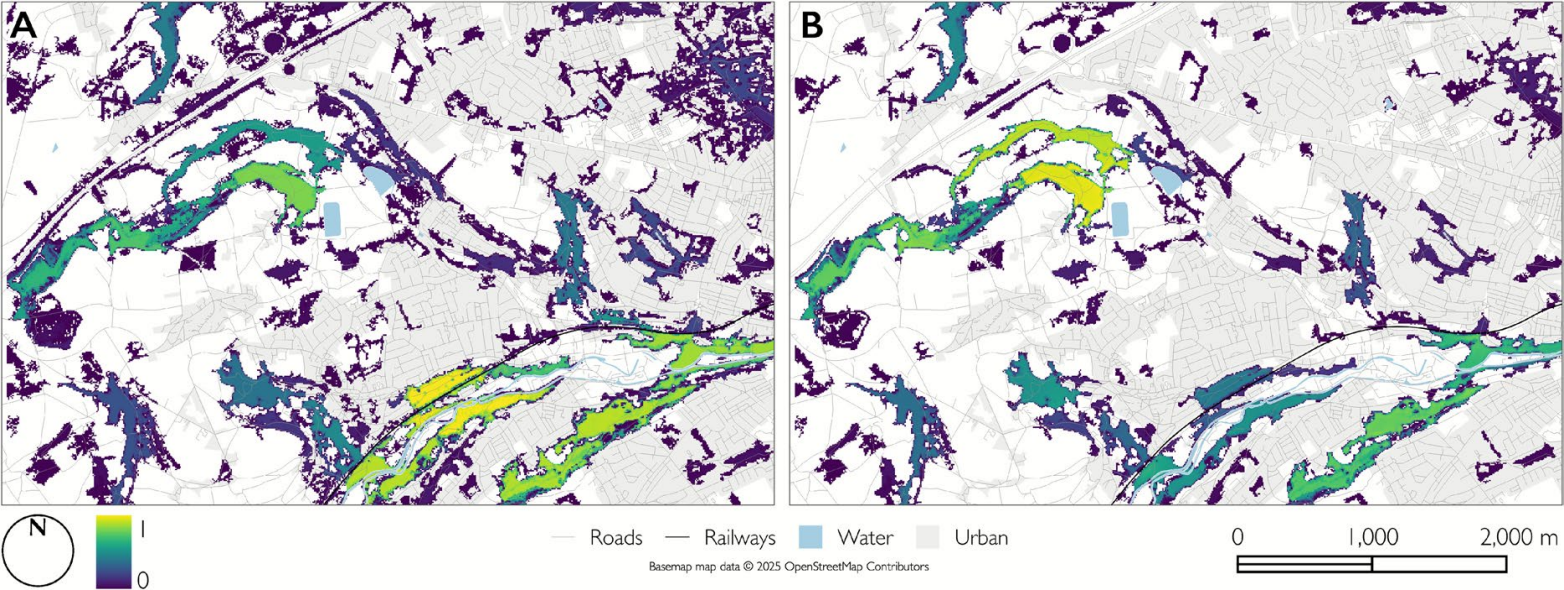
Cell contributions to connectivity, where cell values correspond to $habitat_{c}$ habitat value weighted by patch $dRH$ for all cells arising within habitat patches in the Monte Carlo analysis for **A** the continuous scenario and **B** the functional habitat scenario

Figure 5 shows the spatial distribution of all patches arising in the Monte Carlo simulation where each habitat cell value ($habitat_{c}$ or $fHab_{c}$) has been weighted (at each iteration) by the $dRH$ value of the patch containing that cell. Figure 5A gives the mean of this weighted value (derived from all simulation runs) from the continuous scenario and 5B is the corresponding result from the functional habitat scenario. Each cell’s value therefore reflects an aggregated measure of importance based on its habitat value and contribution to connectivity. The shift in importance from the more urban patches to the South (in areas of higher matrix hostility), to the more rural patches to the West (in areas of lower matrix hostility) is clearly a result of including neighbourhood effects Fig. 5

## Discussion

Our approach offers a method of achieving a functional delineation of habitat that overcomes the limitations of a Boolean view whilst retaining the ability to facilitate the computation of fragmentation-relevant metrics. The case study above illustrates how the approach provides an intuitive reflection of habitat amount, movement cost and connectivity for a focal generic species. This method is therefore the first of its kind that is able to characterize two key distinct but complementary properties of habitat into a metric of overall habitat provision: the morphological transition between habitat patches and the background matrix, and local processes (neighbourhood effects) that contribute to a functional view of habitat. In other words, it addresses the *continuity-contiguity problem* surrounding the habitat patch concept whilst operationalizing neighbourhood effects of nearby cover types. Although other recent approaches have achieved a more functional measure of habitat based on species-specific parameters (e.g. Halstead et al. 2019; Dennis et al. 2024a) such approaches have remained limited with respect to both morphological and functional considerations. For example, although Halstead et al. (2019) leveraged a species distribution modelling approach, they did not formally consider the effect of different biotopes, nor neighbourhood effects, on habitat quality. Similarly, whilst Dennis et al. (2024a, b) operationalised the negative effects of matrix land uses on habitat quality, they did not capture the morphological transition between habitat and non-habitat, considering only functional effects on pre-determined land cover polygons. Moreover, research on fragmentation-biodiversity continues to consider neither local landscape context nor morphological transitions (e.g. Melo et al. 2017; Watling et al. 2020; Watts and Hughes 2024; Riva et al. 2025). Our approach is therefore highly relevant to key current debates in landscape fragmentation research.

### Modelling fragmentation effects on reachable functional habitat

Landscape-level investigations of fragmentation and biodiversity increasingly emphasize the importance of measuring fragmentation *per se*. This correctly asserts the need to consider fragmentation as separate to habitat amount (i.e. testing the effects of fragmentation such that habitat amount is controlled for through study design or statistical procedures). However, this is entirely predicated on a geometric view of fragmentation that does not consider spatial context. In other words, the usual determination of fragmentation assumes a) hard boundaries between habitat and non-habitat and b) that fragmentation relates only to abrupt morphological changes with no consideration of more functional influences such as neighbourhood effects. However, if a resource-based definition and context-driven delineation of habitat is justified (e.g., Dennis 2012; Dennis et al. 2014; Turlure et al. 2019; Hobbs 2024), then it follows that our appreciation of habitat fragmentation, and thus questions around its effect on biodiversity, should consider those same perspectives. For example, in the analysis of our simulation results, the observed relationship between fragmentation (number of patches) and functional connectivity varied substantially between the multivariate contiguous, continuous and functional habitat perspectives (Table 3). The association between number of patches and functional connectivity was negative for the multivariate contiguous (controlling for habitat area) and continuous (controlling for habitat amount) perspectives, but non-significant when observed under the functional perspective of habitat delineation. The absence of a statistically significant relationship is consistent with the lack of consensus within the fragmentation-biodiversity debate and with the view that such a relationship is highly-context specific. Therefore, our approach, leveraging a more functional view of habitat, may shed light on the circumstances in which this relationship is more likely to be positive or negative. This has significant implications for research into the influence of habitat composition and configuration on biodiversity outcomes and we believe that the concept of functional habitat may offer a more helpful perspective on fragmentation itself.

### A fragmented view of habitat amount

A view of fragmentation based on a functional, as opposed to purely geometric, determination presents an opportunity to incorporate important processes relevant to the fragmentation-biodiversity field of inquiry. For example, the functional habitat perspective permits the estimation of neighbourhood effects, leveraging recent methodological developments and foregrounding the role of spatial heterogeneity, a key theme in landscape ecology as yet poorly integrated into fragmentation-biodiversity studies. A view of functional habitat moves us beyond simple measures of contiguous or continuous habitat into resource- and context-based estimates. Thus far, the fragmentation-biodiversity debate has played out under the general expectation that habitat area and amount are commensurate (Evju and Sverdrup-Thygeson 2016; Lindgren and Cousins 2017; Melo et al. 2017; Watling et al. 2020; Zhang et al. 2024). Hence, the effects of fragmentation on biodiversity outcomes have generally relied on a geometric rather than resource-based perspective of habitat provision. Such an assumption does not account for other key characteristics such as habitat complexity or quality. In this respect, degradation is another key driver (in addition to habitat loss) of biodiversity decline (Betts et al. 2022). However, it is rarely considered as a moderator of fragmentation effects but, rather, is typically seen as one of the negative results of fragmentation (e.g. Chase et al. 2020; Hending et al. 2023; Xu et al. 2024). For example, Chase et al. (2020) found biodiversity loss at the patch-level to be a function of ecosystem decay, a process which their study links exclusively to fragmentation effects. Countering this view, Riva and Fahrig (2022) carried out a landscape-level study on the same dataset, demonstrating overall positive effects from increasing fragmentation. However, both studies are limited with respect to the degree to which they control for overall habitat amount: Chase et al. (2020) did not control for habitat amount and hence did not test the effects of fragmentation *per se*, whilst Riva and Fahrig (2022) controlled for only the contiguous habitat area, therefore subjecting their analysis to the *continuity-contiguity problem* (i.e. assuming habitat is equally distributed and of identical quality throughout patches). Hence, these studies suffer from an apparent intractability between habitat area and amount.

This intractability need not persist, however, with the application of our methodological framework. This is because contiguous (area), continuous (amount) and functional (accounting for context) habitat can all be delineated and used with any common fragmentation-related metric (e.g. patch density, size or isolation) through the Monte Carlo approach presented here. We also believe that the relative influence of structural versus functional fragmentation will be a promising avenue of further research using this method. This is because the dominant Boolean approach to estimating fragmentation *per se*, as the number of patches within a given landscape controlling for habitat area, likely reflects neither the number of functional patches nor the amount of functional habitat. The implications of considering a functional habitat approach are underlined by the alternative distributions of $dRH$ (Fig. 5) between “continuous” and “functional” scenarios. Under the continuous scenario, areas of the landscape with the greatest contribution to reachable habitat were allowed to concentrate in areas of relatively high matrix hostility (more heavily urbanised areas to the south, Fig. 5A) whereas, considering the effects of such hostility, the functional scenario resulted in a shift in importance to patches less subject to negative neighbourhood effects (Fig. 5B). This demonstrates that the determination of locations in the landscape contributing most to potential functional connectivity is sensitive to the emphasis placed on neighbourhood effects. Therefore, the appropriate parameterization and modelling of the latter should be a priority when inferring fragmentation effects on functional connectivity.

### Potential theoretical contributions

In terms of operationalizing theoretical aspects of fragmentation-biodiversity research, a focus on neighbourhood effects offers an opportunity to estimate the contribution of surrounding resources. This provides a means to achieve the long-promoted but poorly addressed need to capture a multivariate definition of habitat based on multiple resources as opposed to a single cover type (Dennis and Hardy, 2007; Dennis et al. 2014; Gardner et al. 2024). Related to this, a second opportunity is the ability to formally test the effect of fragmentation on habitat degradation as a function of (negative) neighbourhood effects. Thus, the concept of functional habitat may offer a means to better parameterize the notion of ecosystem decay (Chase et al. 2020). In addition, it provides a more functional route to operationalising the “appropriate ecological distance”, which is central to hypothesis testing on the relative contribution of habitat composition and configuration to biodiversity levels (Fahrig 2013; Watling et al. 2020). This is because the distances employed in the estimation of reachable functional habitat have functional derivations (i.e. they are related to ecological processes). By incorporating foraging and dispersal parameters with resource suitability estimates for focal species, processes that contribute to habitat amount and availability (reachability) are captured. This represents a step-change compared to methods based on Boolean measures of single cover types that ignore the relevance of landscape context.

### Practical challenges to implementation

Though our approach provides an intuitive, functionally-oriented perspective, it depends (as with fragmentation research in general) on the appropriate parameterization of species requirements and behaviour. A major challenge for fragmentation-biodiversity research in general concerns the parameterization of fragmentation-connectivity effects at the community-level. In this respect, our framework has particular synergy with the use of a focal generic species approach (Eycott et al. 2011; Gardner et al. 2024). This is because drawing on a distribution of outcomes permitted through Monte Carlo resampling (e.g. for a focal generic woodland species) allows estimates to be made for species tending towards more specialist or generalist traits. A persistent shortcoming of generic species approaches to estimating parameters for movement and habitat suitability is the use of imperfect estimates whether obtained from the literature or expert consultation. Such estimates provide a limited view and do not consider neighbourhood effects, instead offering a generalized assessment of habitat outside of particular landscape contexts, which limits their applicability. However, the use of such proxies is likely to continue given the practical infeasibility of parameterising entire species pools, their inclusion in computationally expensive modelling techniques and the intractability of subsequent analyses. Though our method also relies on such values, by harnessing uncertainty in the classification of cover types and applying estimates of neighbourhood effects, it nevertheless provides a more comprehensive view of functional habitat. In terms of application to ecological problems concerned with individual species, or groups of species, with similar habitat requirements, it should be possible to leverage the information captured in our approach to estimate a range of potential outcomes. For example, when adopting the focal generic species approach, we can equate different points on the distribution of results to species with similar habitat requirements but varying specialisms and sensitivities to neighbourhood effects. However, it remains to be clarified how working with distributions can be formally linked to species-specific outcomes and this should be a focus of future work. For example, the effective delineation of functional habitat will depend on the ability to model the sensitivity of a given species or group of species to resource quality, diversity and neighbourhood effects. Likewise, the sensitivity of the approach to variability in data resolution and classification schemes will require further testing. Though this remains a key challenge to effective model development, our approach provides a method for explicitly evaluating such sensitivity at scales commensurate with those typical of fragmentation-biodiversity studies.

## Concluding remarks

This paper (along with Dennis et al. this issue) demonstrates an approach to habitat delineation that operationalizes both continuous and contiguous perspectives and a functional understanding of the distribution of habitat resources. The habitat patch remains a central concept informing theory, research and practice within landscape ecology and related themes (Kupfer 2012). The development of metrics that reflect ecological processes continues to be hindered by the limitations of working with, alternatively, contiguous (patch-based) or continuous (gradient-based) approaches (Frazier and Kedron 2017). The methodological approaches described in this pair of papers address this problem via three key developments: 1) rejecting the assumption that habitat “area” is always commensurate with habitat “amount”; 2) operationalizing habitat distributions using *type-2* fuzzy sets and 3) employing spatial kernels to model patch-landscape interactions. Spatial kernels have already been used to delineate edge effects and edge-weight habitat provision (Dennis et al. 2024b) and *type-2* fuzzy sets has been explored in the context of land cover classification (Fisher 2010). The notion of “functional” habitat, such that patch area is not assumed be commensurate with habitat amount due to local heterogeneity, quality or edge effects has also been promoted (Dennis and Huck 2025). We consolidate these related but unintegrated methods and concepts into a single methodology to overcome the need for fixed habitat patch boundaries and to achieve a more flexible delineation of habitat whilst retaining the ability to assess patterns of aggregation. Because this approach harnesses the inherent uncertainty in landscape classification, estimates of habitat area and functional distances are more empirically driven and theoretically supported than previous functionally-oriented attempts to determine habitat provision (e.g. McRae et al. 2008; Saerens et al. 2009; Halstead et al. 2019). This has wide-ranging implications for landscape ecology research due to the increasing relevance of habitat loss and fragmentation on global biodiversity trends. In particular, the single, flexible framework described in this pair of papers advances spatial-ecological methods and permits more rigorous testing of theoretical positions within the fragmentation-biodiversity debate.

## Supplementary Information

Below is the link to the electronic supplementary material.

Supplementary file1 (DOCX 20 KB)

## Data Availability

Data and code to reproduce the analysis will be hosted on the author’s github page upon acceptance at: (https://github.com/dennisMatt/schrodinger).
