# Supplementary material for: Beyond the patch: leveraging functional habitat delineation in fragmentation-biodiversity research
Source: Landsc Ecol. 2026 Jan 13;41(2):37. doi: 10.1007/s10980-025-02290-y (PMC12852187; doi:10.1007/s10980-025-02290-y)
Supplement: Supplementary file 1 — Supplementary file1 (DOCX 20 KB) [file 10980_2025_2290_MOESM1_ESM.docx]

**Beyond the patch: leveraging the notion of realized habitat in fragmentation-biodiversity research**

Dennis, M, Huck, J.J. Holt, D.S. McHenry E., Andersson, E., Sharma, S. and Haase, D.

**Supplementary Materials**

**Table S1** Nesting and foraging suitability values of land cover types used in the study for the focal generic woodland species (from Gardner et al. 2024). Foraging suitability values used in Gardner et al. were on a scale of 0-5 but, for our study, are normalised to a continuous scale of [0,1] to be commensurate with other habitat-related inputs. This was achieved by, for each cover type, taking the peak of its beta distribution for which the alpha and beta parameters are provided in the raw data for the Gardner et al study, hosted by the Centre for Ecology and Hydrology, here: <https://catalogue.ceh.ac.uk/documents/985691ce-e66b-416a-bb85-fc9ad0eca6ed>

| Cover type | Nesting suitability | Foraging Suitability |
| --- | --- | --- |
| Woodland | 0.78125 | 0.826 |
| Grassland | 0 | 0.001 |
| Shrub | 0.43333 | 0.792 |
| Water | 0 | 0.001 |
| Urban* | 0.21111 | 0.187 |

*Values taken from the “Suburban” class in Gardner et al. as best representing the study area

**Table S2** Edge effect and movement cost estimates (taken from Eycott et al., 2011). In addition to the values from Eycott et al. a movement cost of 40 was assigned to the M60 motorway that bi-sected the north-western part of the study area, a six lane highway assumed to incur greater cost that other urban areas.

| **Land cover** | **Movement Cost** | **Edge Effect (m)** |
| --- | --- | --- |
| Broadleaf Woodland | 1.00 | 0.00 |
| Improved grassland | 10.00 | 29.37 |
| Shrub | 2.00 | 0 |
| Freshwater | 10.00 | 0 |
| Urban/built | 5.00 | 75.54 |
|  |  |  |
